# ARCHI: A New R Package for Automated Imputation of Regionally Correlated Hydrologic Records

**DOI:** 10.1111/gwat.13474

**Published:** 2025-02-28

**Authors:** Zeno F. Levy, Robin L. Glas, Timothy J. Stagnitta, Neil Terry

**Affiliations:** ^1^ U.S. Geological Survey, California Water Science Center 6000 J Street, Placer Hall Sacramento CA 95819 USA; ^2^ U.S. Geological Survey, New York Water Science Center 425 Jordan Road Troy NY 12180 USA

## Abstract

Missing data in hydrological records can limit resource assessment, process understanding, and predictive modeling. Here, we present ARCHI (Automated Regional Correlation Analysis for Hydrologic Record Imputation), a new, open‐source software package in R designed to aggregate, impute, cluster, and visualize regionally correlated hydrologic records. ARCHI imputes missing data in “target” records by linear regression using more complete “reference” records as predictors. Automated imputation is implemented using a novel, iterative algorithm that allows each site to be considered a target or reference for regression, growing the pool of complete references with each imputed record until viable gap‐filling ceases. Users can limit artifacts from spurious correlations by specifying model‐acceptance criteria and applying geospatial, correlation, and group‐based filters to control reference selection. ARCHI provides additional functions for visualizing results, clustering records with similar correlation structures, evaluating holdout data, and interactive parameterization with an accessible and intuitive graphical user interface (GUI). This methods brief provides an overview of the ARCHI package, modeling guidelines, and benchmarking on two regional groundwater‐level datasets from the Central Valley, CA and Long Island, NY. We evaluate ARCHI alongside widely used multivariate imputation software to highlight and contextualize its computational efficiency, imputation accuracy, and model transparency when applied to large, groundwater‐level datasets.

## Introduction

Missing data in hydrological records can limit resource assessment, process understanding, and predictive modeling (Gao et al. 2018). Groundwater‐level data are particularly prone to such issues due to the need for distributed measurements across extensive, three‐dimensional aquifer systems that may suffer from sparse continuous‐monitoring locations, sensor failure, or irregular frequency of manual measurements. Complete sets of contemporaneous groundwater‐level records are important for quantifying changes in aquifer storage (Evans et al. 2020a), downscaling remote‐sensing data (Agarwal et al. 2023), calibrating deterministic groundwater‐flow models (Faunt 2009), understanding drivers of compound flood events (Jane et al. 2020), and characterizing water‐resource vulnerabilities to drought (Marchant and Bloomfield 2018; Levy et al. 2021). Therefore, there is broad need to aggregate and impute discontinuous groundwater‐level records sampled at varied frequencies.

Univariate imputation methods leverage information from a single time‐series record and range in complexity from simple linear interpolation to more complicated autoregressive moving average models (Gao et al. 2018; Hamzah et al. 2020). Alternately, multivariate methods attempt to fill missing values at a given monitoring site using exogenous hydrological drivers (e.g., precipitation, soil moisture, pumping) or records from other comparable sites as predictors. Multivariate imputation methods tend to outperform univariate methods when applied to long, contiguous data gaps common to groundwater records (Dwivedi et al. 2022; Bikše et al. 2023).

Prior multivariate imputation studies have also used remote sensing of exogenous hydrological drivers to predict groundwater levels at a regional scale using machine learning (Evans et al. 2020b; Agarwal et al. 2023). While attractive, these techniques require compilation of large hydroclimatic datasets and may propagate artifacts into predictions that were not directly observed in groundwater records (Ramirez et al. 2023). Transfer‐function noise modeling (e.g., Collenteur et al. 2019) is another useful tool for simulating and understanding and the effects of exogenous drivers on groundwater time series but may require compilation of hydrogeologic covariates (e.g., pumping) that are not always available at regional scales. Spatiotemporal interpolation of groundwater‐level measurements is a useful technique to upscale scattered point measurements across distributed monitoring networks (Ruybal et al. 2019; Meggiorin et al. 2023). However, proximate wells may have vastly different head values and dynamics depending on the depth of screened intervals, particularly in areas with large vertical gradients due to pumping (Levy et al. 2021).

A common approach in hydrology is to use linear regression trained on a complete “reference” record to impute a correlated “target” record with missing data (i.e., regression imputation; Gao et al. 2018; Hamzah et al. 2020). Record extension using this general principle has been applied to surface‐flow and groundwater‐level data using techniques that preserve the variance of the target record (Hirsch 1982; Vogel and Stedinger 1985; Dudley et al. 2018) and penalized models that prevent variance inflation and overfitting (i.e., fitting to noise) when using multiple cross‐correlated references (Evans et al. 2020a). However, standard record‐extension methods can be onerous to apply manually to large datasets and require considerable analysis to identify appropriate references for a given set of targets, which is not always feasible when dealing with ubiquitous and irregular gap patterns. Additionally, traditional regression imputation techniques do not account for the underlying uncertainty of the unknown values and tend to underestimate imputation error (Gao et al. 2018; van Buuren 2018; Hamzah et al. 2020).

Multivariate imputations of tabular data with missing‐at‐random gap patterns have been automated using expectation–maximization algorithms that assume a joint multivariate distribution (e.g., the R package Amelia; Honaker et al. 2011) and iterative algorithms provided by other popular R packages, such as MICE (van Buuren and Groothuis‐Oudshoorn 2011) and missForest (Stekhoven and Bühlmann 2012), that apply conditionally specified regression models on a variable‐by‐variable basis. Recently, missForest and other machine‐learning methods have been successfully applied to large groundwater‐level datasets with non‐random, continuous gap patterns (Dwivedi et al. 2022; Bikše et al. 2023; Khampuengson and Wang 2023). Although effective, iterative algorithms and advanced machine‐learning approaches can often be “black box” and difficult to interpret within process‐centered theoretical frameworks (Read et al. 2019). Additionally, automated workflows have not yet incorporated regression methods designed to control the bias‐variance tradeoff (e.g., Hastie 2020) when modeling regional hydrologic datasets with multiple, cross‐correlated predictors. Therefore, there remains a need for automated imputation workflows that can handle large, multicollinear datasets while allowing intuitive identification and suppression of model artifacts by the experienced practitioner.

Here, we present ARCHI (Automated Regional Correlation Analysis for Hydrologic Record Imputation), a new, open‐source software package developed in the R language (Levy et al. 2024a). ARCHI provides an integrated workflow to aggregate regional‐scale monitoring data to a common timestep, automate imputation using established hydrologic record extension techniques, generate non‐parametric prediction intervals using bootstrapping, and evaluate results with interactive graphical tools. Automated imputation is implemented using a novel, iterative algorithm that allows each site to be considered as a target or reference for regression, growing the pool of viable reference sites with each imputation until viable gap‐filling ceases. Users can limit artifacts of spurious correlations by specifying model‐acceptance criteria and applying geospatial, correlation, and group‐based filters to control reference selection. ARCHI provides additional functions for visualizing results, clustering records with similar correlation structures, evaluating holdout data, and interactive parameterization with an accessible and intuitive graphical user interface (GUI).

This methods brief will provide an overview of the functionality of the ARCHI package, focusing on applications to large (hundreds to thousands of sites) groundwater‐level monitoring networks. To this end, we provide examples and guidelines for imputing two datasets of varied correlation structure and dimensionality: (1) annual values for 1394 sites from the southeastern San Joaquin Valley (Central Valley, CA) from 1950 to 2019 and (2) monthly values for 137 sites on Long Island, NY and surrounding coastal mainland areas from 1985 to 2022 (Levy 2024). These two datasets have contrasting ratios of predictors (*p*, sites) to observations (*n*, timesteps) that are used to illustrate advantages and limitations of different regression models. Finally, we benchmark ARCHI on example datasets alongside the widely used R package missForest (Stekhoven and Bühlmann 2012), which employs a similar multivariate imputation algorithm using random forests. Benchmarking results and further discussion of other widely used multivariate imputation software serve to highlight and contextualize ARCHI's computational efficiency, imputation accuracy, and model transparency when applied to large, groundwater‐level datasets.

## Software Overview and Workflow

The following section provides an overview of the ARCHI package capabilities and workflow using example datasets. The software is publicly available for download at: https://code.usgs.gov/water/ARCHI/. The software release contains a README file in markdown format that is rendered on the landing page and includes installation instructions and a detailed workflow with code examples using the NY dataset, which is included with the package. Source code for individual package functions included in the 1.0.0 release and described below can be viewed and downloaded from the online repository at: https://code.usgs.gov/water/ARCHI/‐/tree/1.0.0/R.

### Pre‐Processing

Groundwater levels and other hydrological measurements may be sampled on vastly different timescales and frequencies. ARCHI allows users to aggregate measurements of varied periodicity to a common timestep (daily, weekly, monthly, seasonal, or annual) using the “timestep_grid()” function. The function intakes date‐ and site‐indexed observation values in a long‐format table (one row for each observation value; example input file format can be found at: https://water.code‐pages.usgs.gov/ARCHI/reference/LI_data.html) and aggregates them to a wide‐format “grid” where rows represent sequential timesteps and columns represent unique sites. Observation values falling within a discrete timestep (e.g., calendar year) can be aggregated by mean, median, minimum, maximum, or user‐specified quantile. Timesteps containing no observation values for a given site are populated with NAs (R syntax for a “not available” or “missing” value), which are recognized in subsequent process steps as candidates for imputation. The output grid includes a leading “timestep” column containing calendar dates, which are used to index model timesteps for ease of plotting or combining with observation dates. Timesteps can be indexed by their first or median calendar date.

The “timestep_grid()” function affords considerable flexibility in defining timesteps, including user‐defined water years and seasons. An additional month filter allows selective aggregation of data observed in a subset of calendar months. Flexible data discretization can aid in defining imputation targets. Daily timesteps may be more appropriate for continuous monitoring data, whereas seasonal or annual timesteps may be better for more irregularly monitored sites. ARCHI does not currently grid on sub‐daily timesteps (e.g., hours, minutes, seconds) but will impute user‐formatted grids of such data without a leading timestep column, indexing timesteps in model output by sequential integers. As a final pre‐processing step, the “trim_grid()” function allows the user to filter the timestep grid by record completeness, remove timesteps without any observation values, and remove near‐zero variance records. The generalized, tabular structure of the timestep grid can be input to other popular R packages for multivariate imputation such as missForest, MICE, or Amelia after removing the leading “timestep” column.

Data pre‐processing requirements vary by dataset. Here, we illustrate ARCHI workflows for two contrasting datasets from Central Valley, CA and Long Island, NY (Levy 2024). The CA dataset includes data from 4381 wells within an 8500 km^2^ subregion of California's Central Valley known as the southeast San Joaquin Valley. The NY dataset includes data from 476 wells within the 3630 km^2^ extent of Long Island, NY, and surrounding mainland sites in NY and CT. Full descriptions of data sources and site‐selection criteria are available in the data release of Levy (2024).

The CA dataset consists of groundwater‐level measurements of varied frequency, but most often having only 1–2 measurements per year, during September–October and January–February, which represent periods of most pronounced drawdown and recovery, respectively (Figure S1a). We aggregated annual median “spring” groundwater levels (January–April) for the CA dataset to focus on interannual variability as described by Levy et al. (2021). In contrast, the measurements in the NY dataset are spread more evenly throughout the year (Figure S1b). In this case, we aggregated monthly medians to better define seasonal variability of groundwater levels. Each dataset was further filtered to select sites with sufficient data for imputation. Only sites with at least 50% and 35% of timesteps containing observations were retained for imputation of the respective CA and NY datasets (see “Modeling Guidelines” section for further discussion). This resulted in 1394 sites with at least 35 out of 70 annual spring medians for CA and 137 sites with at least 202 out of 576 monthly medians for NY (Table 1).

**Table 1:** Select Features of Example Input Datasets and Attribution of Values Imputed Using ARCHI Default Settings.

					Percent of Imputed Values Filled by			
Dataset	Sites (*p*)	Timesteps (*n*)	Percent Missing Values (NAs)	Mean Standard Deviation of Observed Site Records, in Meters	Interpolation	Extrapolation	Gap_fill	Extension
Central Valley, CA	1394	70	32	5.8	73	27	27	73
Long Island, NY	137	576	39	0.6	98	2	56	44

**Figure 1:**
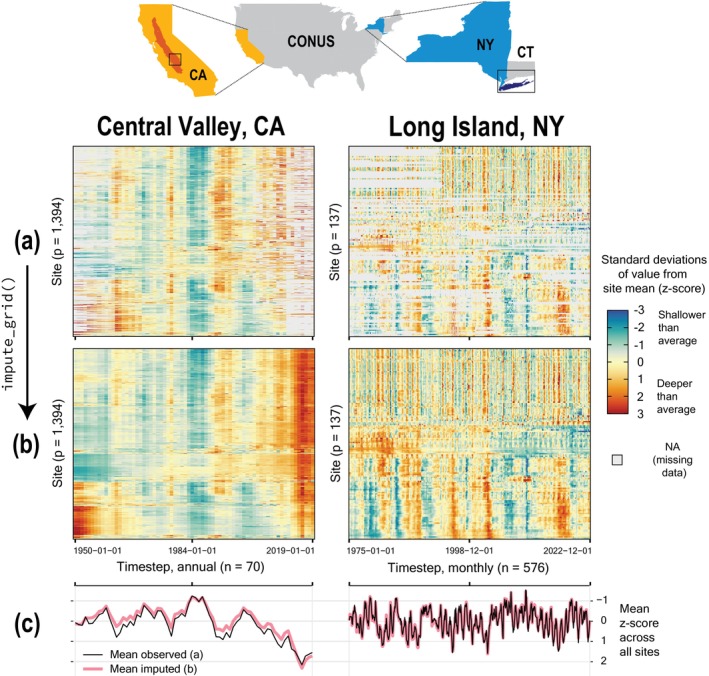
Location of study regions in the conterminous United States (CONUS; top) indicated by rectangular bounding boxes within state inset maps. The full extent of the Central Valley in California (CA) and Long Island in New York (NY) are highlighted by darker shading within respective state maps. The lateral extents of bounding boxes for respective study regions are 133 and 196 km for CA and NY (the latter of which also includes a portion of coastal Connecticut [CT]). (a) Observed and (b) imputed example groundwater‐level datasets from the CA (left) and NY (right) study regions are shown with heatmaps below respective state maps. Heatmaps represent *z*‐score standardized groundwater‐level records ordered along the *y*‐axis by hierarchical clustering of imputed records. (c) Time series of *z*‐scores averaged across timesteps for observed and imputed datasets.

The “grid_heatmap()” function can be used to compare missingness patterns and relative groundwater‐level fluctuations between the two datasets (Figure 1a). Although the two datasets have contrasting dimensionality, they both have similar proportions of missing values (32% for CA and 39% for NY; Table 1). However, the CA dataset has more missing values at the beginning and end of the period of interest, whereas missing values are spread more evenly throughout the NY dataset. Broad regional trends in the two datasets can be further evaluated by visualizing depth to groundwater measurements as standard deviations from the site mean (*z*‐score) and averaging those values across each timestep (Figure 1c). Most of the variability in the CA dataset comes from interannual changes driven by increased groundwater pumping during drought superimposed over persistent groundwater‐level decline during the past 30 years (Levy et al. 2021). In contrast, the NY dataset is dominated by seasonal and interannual variability during dry and wet periods with fewer decade‐scale trends (Busciolano 2005).

### Imputation

#### Algorithm Overview

The “impute_grid()” function implements a novel, iterative algorithm to impute NAs within a tabular grid of observation values where columns represent unique site records that are row‐indexed to common timesteps. The algorithm has a similar form to that of MICE and missForest which, given the set of variables (*P*_1_, *P*_2_, *P*_3_, …, *P*_*i*_), sequentially impute missing values in *P*_1_ by regression on (*P*_2_, *P*_3_, …, *P*_*i*_), missing values in *P*_2_ by regression on (*P*_1_, *P*_3_, …, *P*_*i*_), missing values in *P*_3_ by regression on (*P*_1_, *P*_2_, …, *P*_*i*_), and so on. Variable‐by‐variable multivariate imputation strategies of this nature avoid overt specification of a joint multivariate distribution for the entire dataset (e.g., Honaker et al. 2011) and are commonly referred to as fully conditional specification (van Buuren 2007), chained equations (van Buuren and Groothuis‐Oudshoorn 2011), or sequential regression (Raghunathan et al. 2001).

**Figure 2:**
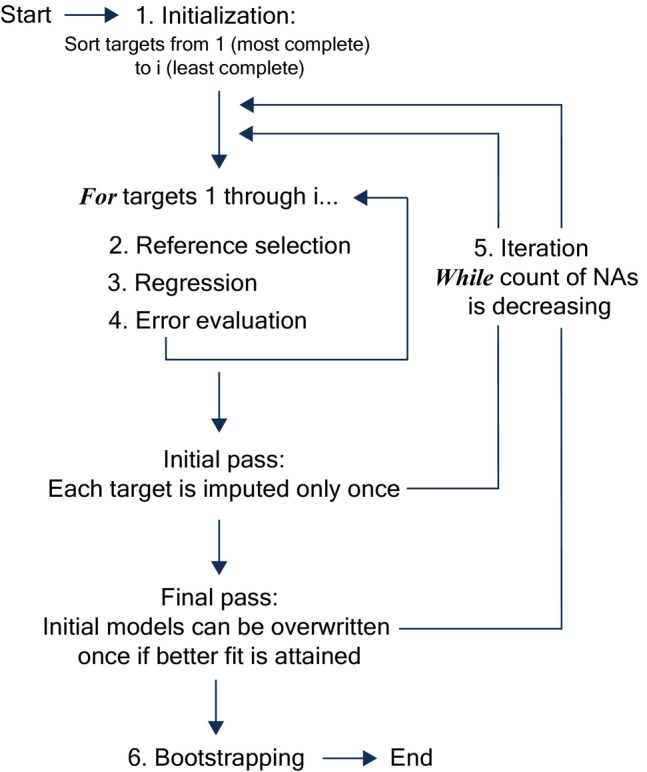
Simplified process diagram of ARCHI's imputation algorithm called by the “impute_grid()” function.

The ARCHI algorithm proceeds in the manner described above from the most to the least complete variables (herein, “targets”), training regression models on a selected subset of correlated variables as predictors (herein, “references”). Missing values are only imputed if regression models satisfy a user‐specified error threshold, and the target sequence is cycled through iteratively until viable gap‐filling ceases. The ARCHI algorithm has six primary stages: (1) initialization, (2) reference selection, (3) regression, (4) error evaluation, (5) iteration, and (6) bootstrapping (Figure 2). Here, we describe the basic functionality of algorithm components, contextualize with discussion of other imputation algorithms, and apply the algorithm to example datasets. Full documentation of the “impute_grid()” algorithm is available in the software release of Levy et al. (2024a) and can be viewed online at: https://water.code‐pages.usgs.gov/ARCHI/reference/impute_grid.html.

#### Initialization

Similar to missForest and other sequential regression approaches (e.g., Raghunathan et al. 2001), ARCHI initiates a target imputation order by sorting sites from most to least complete. Prior variable‐by‐variable imputation algorithms of this nature have required complete cases to establish regression models, which are often estimated during algorithm initialization. For example, missForest and MICE will make an initial estimate to fill the NAs for a given variable using its mean or a random sample of its observations, respectively, which are iteratively overwritten using predictive models trained on the remaining variables. This ensures complete cases for regression but dilutes correlations and requires determination of an appropriate number of iterations to achieve convergence, which may not be quantifiable (van Buuren 2018). Conversely, ARCHI makes no initial guess to fill NAs and instead only imputes missing values based on the output of regression models with reasonable fits to the observed data.

The above requirement puts a high premium on having abundant complete or near‐complete records to “seed” initial regression models used to grow the pool of potential references. To aid in this, ARCHI allows creation of synthetic references from composite site records during initialization with the “add_std_means” parameter. The default mode (“add_std_means = TRUE”) creates a synthetic, complete reference from the mean of all *z*‐score standardized site records (“mean observed” in Figure 1c) representing a relative, regional groundwater signal. If categorical groups are assigned to sites in the input metadata, those group means are added as additional predictors. Such groupings can be used to generate synthetic groundwater records representative of specific aquifer areas or depth zones. This novel feature allows initial models to be trained on predominant signals in the dataset as opposed to records artificially populated with the static site mean or a random sample.

#### Reference Selection

ARCHI identifies and culls a pool of potential references for each target based on (1) matching datapoints, (2) correlation to the target, (3) optional “cutoff” filters, and (4) model parsimony. This differs from MICE and missForest, which impute targets by building a model utilizing all remaining sites. Flexible reference selection allows models to be trained on meaningful subsets of available predictors.

ARCHI requires all potential references have timestep‐indexed datapoints (observed or imputed) corresponding with all NAs in the target record, ensuring complete imputations. References must additionally have sufficient matching datapoints to evaluate correlation with the target and train a regression model. The proportion of matching datapoints required by the reference is controlled by the “relax” parameter. The default value of “relax” is 0.1, which requires references to have observations corresponding with at least 90% of the target record. For example, a target record with 100 observations and 50 NAs would require references to have at least 90 corresponding observations to train a regression model and an additional 50 to predict all missing values. This allows use of partially complete references that leverage a critical mass of target observations to train models. ARCHI implements a minimum data threshold and will not impute records with <10 observations.

References that meet data requirements are then tested for correlations to the target. Correlation is evaluated using the Pearson product–moment correlation coefficient (herein, Pearson's *r*) to assess linear associations between time series. Only references that have significant correlations to the target with *p*‐values ≤ 0.05 are retained for use in regression models. Additional optional “cutoff” filters can be implemented that limit reference selection to those within a geospatial buffer distance from the target site (e.g., Ramirez et al. 2023), minimum Pearson's *r* value (e.g., Feng et al. 2014), or membership in the target's pre‐assigned “group” (e.g., Raghunathan et al. 2001). “Group” is an optional, user‐defined categorical variable that can be used to insulate targets from references lacking comparable site characteristics such as location in a specific aquifer area or depth zone (e.g., “shallow” vs. “deep”). Geospatial‐ and group‐based cutoffs require inclusion of site metadata containing coordinates and user‐specified group assignments (example site metadata format can be found at: https://water.code‐pages.usgs.gov/ARCHI/reference/LI_sites.html). Such controls allow conditional specification of regression models on meaningful sub‐populations of related sites as opposed to assuming a joint multivariate distribution for the entire dataset.

The resultant pool of potential references may be further culled to control model parsimony, which can enhance interpretability and avoid overfitting. The user may specify the maximum number of references with the “n_refwl” parameter. Setting this parameter to a positive integer selects the top “n_refwl” most correlated references (greatest Pearson correlations to the target) and setting to “max” allows use of all potential references. The number of references can be further limited based on the number of training observations. The “p_per_n” parameter allows the user to specify the maximum number of model predictors (*p*) as a fraction of the number of training observations (*n*). For example, setting “p_per_n” to 0.1 removes least correlated predictors until a model can be established that has no more than one predictor per every 10 observations. ARCHI removes least correlated predictors before fitting until the condition *p* < *n* is met for all regression models.

#### Regression

References meeting selection criteria are used as predictors to train a regression model with the target record as the dependent variable. ARCHI provides three methods to fit linear models: (1) ordinary least squares (OLS), (2) ridge regression (ridge), and (3) maintenance of variance extension, type 1 (MOVE.1). The OLS method uses the standard linear model available in the R “stats” package (R Core Team 2021). Ridge regression, also known as L2 regularization, is a form of linear regression that avoids overfitting through addition of a penalty term to the linear model (Hastie 2020). The effect of the penalty term on the model is controlled by the hyperparameter *λ*. ARCHI uses the R “glmnet” package for fitting ridge regression models with automated selection of *λ* using cross‐validation (Friedman et al. 2010). The penalty term prevents variance inflation in the presence of multicollinearity by reducing model variance in exchange for a slight increase in bias. Alternately, the MOVE.1 model, also sometimes referred to as “the line of organic correlation,” preserves the mean and variance of the target record (Hirsch 1982). MOVE.1 is preferable for groundwater applications to the MOVE.3 model, which is commonly used to extend annual peak surface‐water flow records (Vogel and Stedinger 1985; Granato 2009; Siefken and McCarthy 2022). This is because MOVE.3‐type models are not recommended for time series with high degrees of autocorrelation (Matalas and Jacobs 1964), a common characteristic of groundwater‐level records. ARCHI uses the implementation of MOVE.1 described by Hirsch (1982), which is fit using a single predictor variable. ARCHI selects the reference with highest correlation to the target to fit MOVE.1 models.

Training data for all regression models are range‐normalized between 0 and 1 before fitting, as described by Evans et al. (2020a). Model predictions are subsequently back transformed to the original observation units in the output. MICE similarly allows flexibility to use different predictive models within its iterative algorithm, but does not currently offer MOVE‐type models or ridge regression with automated fitting of *λ* by cross‐validation.

#### Error Evaluation

ARCHI accepts regression models for imputation based on their ability to fit the observed data. The fits are computed using all observed data points to which a corresponding simulated value has been computed for each site‐wise regression. Error evaluation within the algorithm is based on standard metrics of residual error for individual linear regression models and represents model training error using all available data. Random holdout data for model validation can be sampled prior to implementing the algorithm using other functions included in the ARCHI package (see “Exploration and Evaluation” section for details).

Model fits are evaluated against a user‐specified error threshold (“error_thresh”). The Nash‐Sutcliffe efficiency (NSE) is the default error metric (“error_method”) because it is normalized to the mean of observed values and therefore generalizable to diverse datasets of varied scales. The NSE is defined as:

(1) $$\mathrm{NSE}=1-\frac{\sum_{i=1}^{N}{\left(S_{i}-O_{i}\right)}^{2}}{\sum_{i=1}^{N}{\left(O_{i}-\bar{O}\right)}^{2}}$$

where N represents the number of observed values, $S_{i}$ represents the *i*th simulated value, $O_{i}$ represents the *i*th observed value, and $\bar{O}$ represents the mean of observed values. The NSE ranges from −∞ to 1, with 1 representing a perfect model fit and values below 0 indicating the model is a worse predictor than the mean of the observed data (Nash and Sutcliffe 1970). The default setting uses an NSE threshold of 0, signifying models must not perform worse than the mean of target observations (i.e., NSE < 0) to be accepted for imputation. Additional model‐evaluation metrics include mean absolute error (MAE), root mean square error (RMSE), and variance‐normalized root mean square error (NRMSE; see R package documentation for details, available online at: https://water.code‐pages.usgs.gov/ARCHI/reference/impute_grid.html). For these metrics, models must not have error rates exceeding the specified threshold to be accepted for imputation.

Implementation of an error threshold is a novel feature of ARCHI in the context of multivariate imputation algorithms. Similar algorithms such as MICE and missForest are agnostic to model fits and will impute records regardless of predictive accuracy. The error threshold approach prevents propagation of model artifacts through the dataset and can help identify anomalous records that are not well explained by regional time‐series dynamics.

#### Iteration

Imputed records are retained and used as candidate references for subsequent imputations. An iteration is complete after imputation has been attempted once for all targets. Remaining unimputed targets are cycled through from most to least complete in subsequent iterations until all NAs are filled or satisfactory regression models can no longer be fit to the observed data. ARCHI, by default, initiates an optional final pass after all potential gaps have been filled wherein initial imputations are overwritten if an improved fit can be obtained using the fully imputed dataset. The final pass can improve imputation accuracy but is prone to data leakage, which can limit quantification of model uncertainty (see “Model Evaluation and Benchmarking” section for details).

Alternatively, MICE and missForest overwrite imputations during each iteration and require specifying the number of model iterations or a stopping criterion. This can increase computation times with diminishing returns. Multiple iterations are needed for these models to ensure artifacts of initial guesses are sufficiently attenuated by regression. Because ARCHI makes no initial guesses at the unknown values and resists imputation based on spurious model fits, minimal overwriting of initial models is required.

#### Bootstrapping

Non‐parametric prediction intervals can be generated for ARCHI models using the bootstrapping method of Mougan and Nielsen (2022), which makes no assumptions about the sampling distribution of the data or noise. The bootstrap is implemented at the end of the imputation routine by randomly sampling the final training dataset for each target's regression model “B” times with replacement. The selected regression model is fit to the resampled training data and used to generate a distribution of residuals weighted by the bootstrapped model's relative overfitting rate. By accounting for overfitting, the method of Mougan and Nielsen (2022) can be applied to a wide variety of parametric and non‐parametric regression models, including machine learning applications. The distribution of the weighted residuals is used to define the bounds for the prediction interval based on a user‐selected alpha value (e.g., default “alpha” is set to 0.05 for generation of 95% prediction intervals). We suggest a default of 500 for B, which can be adjusted by the user as needed.

MICE and missForest utilize different approaches to quantifying imputation error. MICE is a multiple imputation approach that generates *m* (typically five or more) imputed datasets originating from *m* random initializations. This results in *m* estimates for each missing value, which can be pooled to evaluate parameter error in various statistical applications (van Buuren 2018; Little and Rubin 2019). Alternatively, missForest is a single imputation technique (only one imputed dataset is returned) that addresses uncertainty by averaging over a random, bootstrapped sample of unpruned regression trees and producing an out‐of‐bag (OOB) error estimate derived from the residuals for the aggregate dataset and individual variables (Stekhoven and Bühlmann 2012). ARCHI is also a single imputation model that produces a deterministic “best estimate” imputed dataset. ARCHI's bootstrapping approach to error estimation provides prediction intervals that can be used to bound the uncertainty of modeled time series and identify outliers.

#### Application to Example Datasets

We imputed both CA and NY datasets using the “impute_grid()” default settings: ridge regression with an NSE error threshold of 0 using the maximum number of references constrained by a “p_per_n” ratio of 0.5. Heatmaps of the observed (a) and imputed (b) datasets are compared in Figure 1. The observed and imputed *z*‐scores have similar values when averaged across sites (Figure 1c), indicating the imputation preserved the central tendency of the observation data for individual timesteps across the period of interest.

### Exploration and Evaluation

Output from the “impute_grid()” function can be saved as an “ARCHI” object in R and contains a variety of model output and diagnostics. In addition to providing the imputed dataset, modeled values, and prediction intervals in wide “grid” format, results are also compiled in long format with observed and imputed values row‐indexed by site and date. Modeled values can be directly compared with input observations to evaluate training error, which can be visualized for individual time series using the “ts_plots()” and “resid_plots()” functions. Output also contains additional metadata describing whether imputed values are either within (“interpolation”) or outside (“extrapolation”) the range of the training data and are additionally within (“gap_fill”) or outside (“extension”) the period of the training data. For example, we can see that imputation of the CA dataset required considerably more extrapolation and extension beyond the bounds of the training data compared to that of NY (Table 1). A “model_stats” summary table compiles error‐based fit metrics used for acceptance thresholds along with an additional performance statistic, the variance ratio, which equates to the variance of simulated values divided by the variance of corresponding observations for each site. Variance ratios <1 indicate a loss of variance for modeled compared to observed values at a given site. The variance ratio is equivalent to the NSE for OLS models because they preserve the mean of the observation data.

ARCHI provides several additional functions for visualizing, clustering, and evaluating model results. The “grid_heatmap()” function allows for visual intercomparison of observed and imputed datasets (Figure 1a and 1b). Sites may be ordered along the *y*‐axis by proportion of missing values (completeness), or hierarchical clustering on Pearson correlation distances as described by Levy et al. (2024b). Site orders using these two sorting methods can be exported and passed to another heatmap for direct intercomparison of input and imputed datasets. For example, Figure 1 was generated by clustering the complete records for the imputed dataset (Figure 1b) and then using the resultant *y*‐axis site order to make a similar heatmap with the un‐imputed input dataset (Figure 1a). The “cluster_grid()” function can be used to attribute sites by cluster group and plot respective standardized group means as time series. The number of clusters (*k*) can be user specified or automatically optimized by the silhouette index as described by Levy et al. (2024b). These methods allow the user to rapidly identify predominant patterns in regional time series and group sites with similar correlation structures.

The “hold_grid()” and “hold_eval()” functions allow the user to randomly withhold a specified proportion (p) of observation values from the input grid to evaluate model performance. The random sample of holdout observations is returned with corresponding imputations along with a summary RMSE or NRMSE value. Here, we evaluate holdout performance by NRMSE, defined as:

(2) $$\text{NRMSE}=\sqrt{\frac{\frac{1}{N}\sum_{i=1}^{N}{\left(S_{i}-O_{i}\right)}^{2}}{\sigma_{o}^{2}}}$$

where N represents the number of observed values, $S_{i}$ represents the *i*th simulated value, $O_{i}$ represents the *i*th observed value, and $\sigma_{o}^{2}$ represents the variance of the observed values. The NRMSE represents the model's root mean square error (RMSE) as decimal fraction of the standard deviation of observed values. The RMSE represents model error in the original measurement units and can be calculated by removing the denominator from Equation 2. Comparison of RSMEs or NRMSEs for random holdouts can be used to assess the relative performance of various imputation models but should not be used to infer imputation uncertainty (Oberman and Vink 2023). Model inference can be assessed by supplying bootstrapped prediction intervals to the “hold_eval()” function to compute a coverage rate, defined here as the proportion of withheld observations within corresponding prediction intervals.

**Figure 3:**
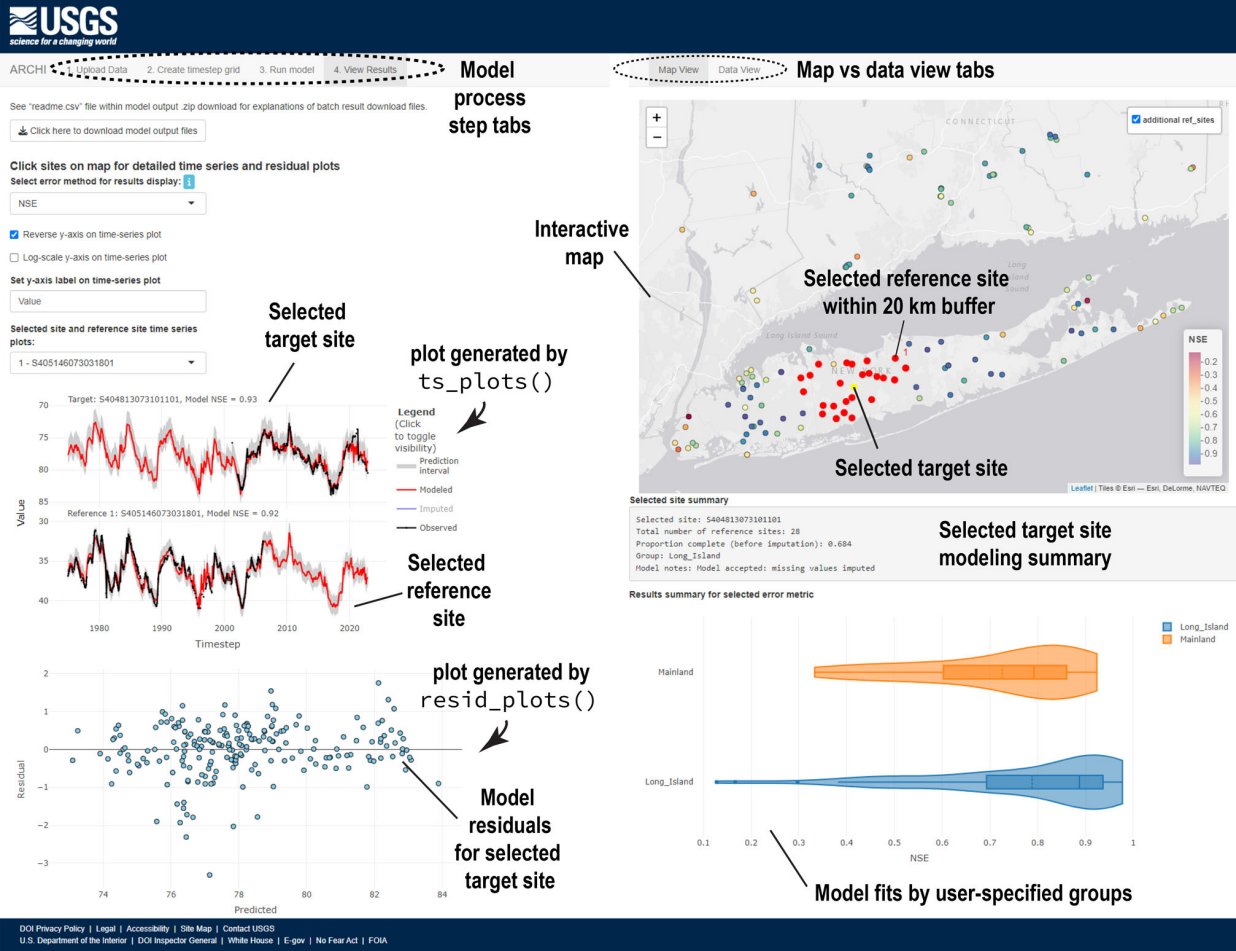
Screenshot from ARCHI's graphical user interface (GUI) showing interactive map for exploring modeling results. Results for the NY dataset are shown after imputation using ridge regression with a 20 km geospatial buffer to constrain automated reference selection around targets.

ARCHI has a GUI built using the R “shiny” package (Chang et al. 2023). The GUI can be run after the ARCHI package has been loaded in R with the single line of code via the “run_gui()” function. The GUI provides a flexible, interactive interface for implementing the ARCHI workflow, evaluating model results, and adjusting model parameters in real time. The GUI allows exploration of imputation results in “data view” using grid_heatmap() plots or “map view” where model fits and associated references for individual sites can be explored using an interactive map that automatically updates graphics generated from “ts_plots()” and “resid_plots” functions (Figure 3). Model results can be downloaded to a .zip file, which includes a data dictionary detailing contents of output files. The GUI allows for intuitive evaluation of hydrologic model results in geospatial context and expands the ARCHI user base to practitioners who may not be fluent in R.

## Modeling Guidelines

### How Many Datapoints?

Targets with high missingness proportions could have spurious correlations with potential references yielding unreliable imputations. There is no one‐size‐fits‐all method for determining how many datapoints are required for successful imputation of a target record. Additionally, the structure of the missingness patterns can exert strong controls on imputation accuracy. Dwivedi et al. (2022) used an information entropy approach to show accurate imputations could be achieved for groundwater‐level records with up to 90% missing values occurring at random intervals as opposed to up to 50% for block (contiguous) intervals. Imputation accuracy may decrease if missing data occurs during hydrological extremes (Dwivedi et al. 2022; Bikše et al. 2023), which can induce non‐random data gaps related to sensor failures that are more likely to occur during droughts or floods.

The most direct approach to estimate a minimum data threshold for reliable imputation is to identify representative sites with near‐complete records and induce synthetic gaps in the data at different patterns and missingness rates. We provide an example of such a simulation in Figure S2. Identification of typical‐gap patterns in potential targets can help refine simulation designs (Bikše et al. 2023). Such simulations can better define a minimum data missingness threshold for successful imputations. However, results of such simulations are dataset specific and comprehensive assessment of typical‐gap patterns for the example datasets presented here were outside the scope of current study.

We recommend, as a general starting point, only imputing records that are at least 50% complete, and subsequently adjusting this value so target records have adequate data to train models across the range of observed hydrologic conditions (**Guideline 1**). We lowered the data threshold to 35% for the NY dataset, which represents a minimum of about 16 years of monthly observations and appears to provide training coverage across a range of seasonal‐to‐interannual climate conditions. Ultimately, it is up to the practitioner to determine the minimum data threshold required to meet study goals. ARCHI provides a flexible platform for rapid assessment of imputation results at different missingness rates.

### Setting the Error Threshold

ARCHI is unique compared to other multivariate imputation programs in that it implements an error threshold to evaluate the suitability of a regression model for imputation based on fit to the observed data. The default NSE error threshold of 0 provides a permissive lower limit, only excluding exceptionally poor model fits with little predictive power. From there, we encourage the user to gradually increase the NSE threshold and examine model output. More stringent error thresholds will tend to result in fewer imputed records. We recommend tightening error thresholds until a major drop‐off in success rate (proportion of targets successfully imputed) occurs (**Guideline 2**). For example, both NY and CA datasets were able to impute nearly all (>98%) of the target records using an NSE threshold of 0.4 with diminishing returns at higher values (Figure S3). Further tightening of the error threshold depends on user‐defined modeling criteria. Some studies suggest an NSE of 0.5 or greater provides a satisfactory modeling objective for a variety of hydrological applications (Moriasi et al. 2007; Dudley et al. 2018).

Fundamentally, more stringent error thresholds can limit artifacts of bad model fits propagating through datasets but can also result in information loss whereby fewer complete records are available to train subsequent models. The ARCHI GUI can be used to interactively adjust the error threshold and gain an intuitive understanding of its effects on model output. Other included error metrics (MAE, RMSE, and NRSME) can also be used to meet various modeling objectives.

### Predictor Selection

**Figure 4:**
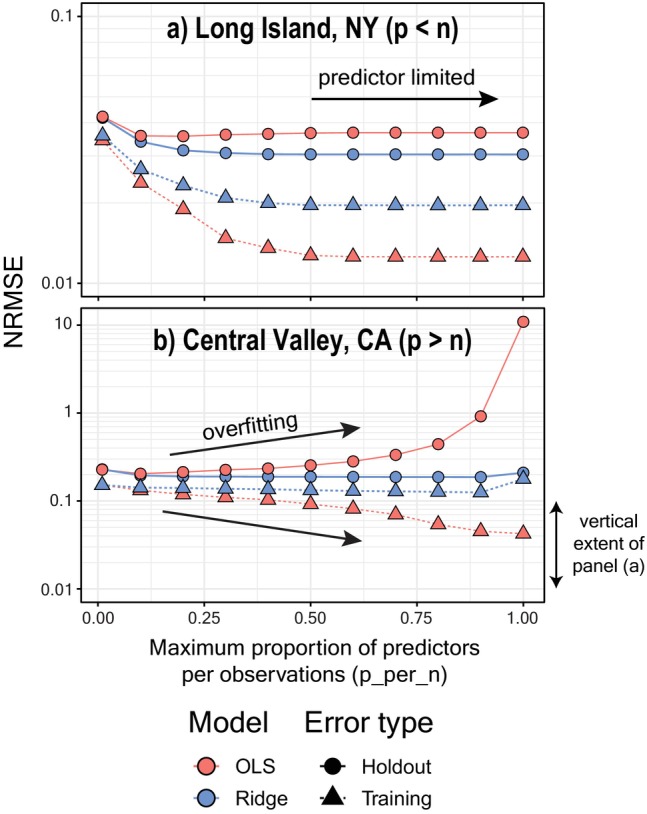
Model error (NRMSE) plotted as a function of p_per_n parameter for (a) NY and (b) CA datasets. The lowest tested p_per_n value was 0.01 with subsequent values ranging from 0.1 to 1.0 by increments of 0.1 (Levy 2024). Training error represents NRMSE values for all modeled versus observed values computed for each complete dataset. Holdout errors are the mean of 200 random holdouts evaluated using 5% of the original observation data. The *y*‐axes are plotted on a log‐scale and vary in range between the two panels to highlight effects of the p_per_n parameter on model results and not intercomparison between the two datasets.

ARCHI allows the user to select a maximum number of predictors used in regression models as a positive integer (e.g., top 10 correlated reference sites) or as a proportion of the number of training observations (p_per_n). A larger number of predictors can enhance the model's capability to fit to the observed data at the risk of overfitting to noise or overwriting local dynamics with highly regionalized signals. Overfit models tend to overperform on training data and underperform on holdout data (Mougan and Nielsen 2022). We used our example datasets to compare training and holdout errors for both OLS and ridge models across a range of p_per_n ratios (Figure 4). Increasing the p_per_n ratio decreased holdout and training errors for both models using the NY dataset, which is predictor limited (*p* < *n*) and therefore less prone to overfitting. However, we found OLS models of the high‐dimensional (*p* > *n*) CA dataset were vulnerable to overfitting as p_per_n exceeded 0.1 and approached unity, indicated by large increases in holdout error coinciding with decreased training error.

The ridge model, by contrast, performed similarly across a range of p_per_n ratios due to its ability to handle multicollinear data and resist overfitting. A slight increase in both training and holdout errors occurred for ridge models as p_per_n approached unity. This effect is likely due to over‐regularization as the penalty parameter is automatically adjusted to combat variance inflation when increasing numbers of correlated predictors are added to the model. We recommend constraining reference selection using p_per_n values of 0.5 (one predictor for every two observations) for ridge and 0.1 (one predictor for every 10 observations) or less for OLS to avoid over‐regularization and overfitting, respectively (**Guideline 3**). Faster and more interpretable models may be achieved by setting the maximum number of references (“n_refwl”) to integers ≤10.

## Model Evaluation and Benchmarking

Here, we use the two example datasets to evaluate the capacity of ARCHI models (OLS, ridge, and MOVE.1) to fit the observed records and benchmark using holdout datasets alongside the widely used R package, missForest. The missForest package employs a single imputation algorithm, which provides comparable output to that of ARCHI as opposed to multiple imputation methods. Stekhoven and Bühlmann (2012) found missForest had high imputation accuracy relative to other single imputation methods (*k*‐nearest neighbors and missingness pattern alternating lasso) and a multiple imputation method (MICE) when applied on diverse biomedical datasets. The missForest algorithm has also successfully been applied on a variety of hydrological datasets with high imputation accuracy relative to other common methods such as univariate linear interpolation, principal component analyses‐based models, and MICE (Feng et al. 2014; Arriagata et al. 2021; Bikše et al. 2023). Benchmarking these software programs alongside each other is not meant to provide conclusive evidence of relative imputation accuracies, but rather to evaluate whether comparable results may be achieved following modeling guidelines set forth in respective companion papers.

For ARCHI models, we modified default settings with an NSE error threshold of 0.4, which effectively balances enhanced restriction of models with high success rates (as per **Guideline 2**) for both example datasets and allows for better intercomparison of results with non‐exclusionary models such as missForest. We set p_per_n ratios of 0.1 and 0.5 for OLS and ridge models, respectively (as per **Guideline 3**). Results are presented for the default final pass models unless otherwise noted. We used the default settings for missForest (ntree = 100, mtry = √p), which Stekhoven and Bühlmann (2012) recommend can be used on diverse datasets without the need for tuning or cross‐validation.

### Goodness‐of‐Fit Evaluation

**Figure 5:**
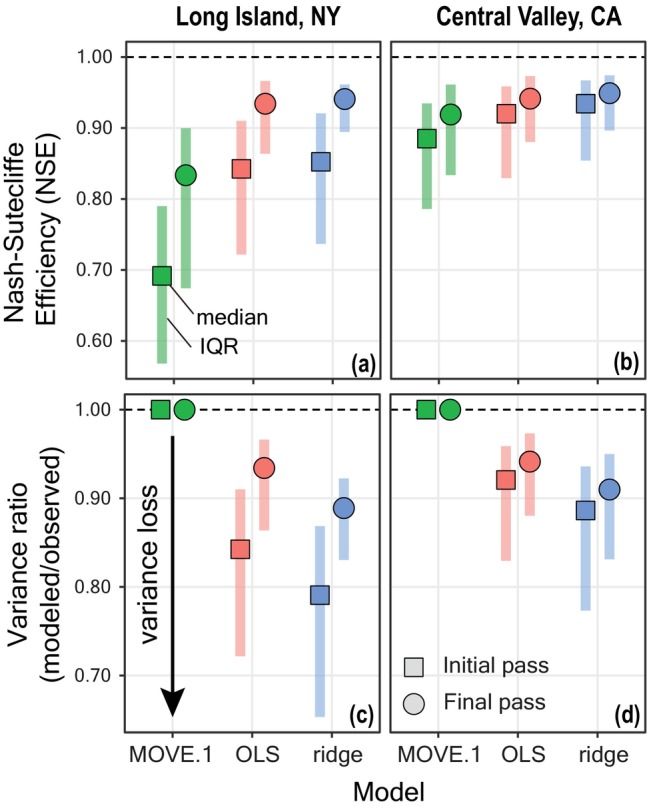
Goodness‐of‐fit metrics for site records imputed using ARCHI on full NY (left) and CA (right) datasets. (a, b) Nash‐Sutcliffe efficiencies and (c, d) variance ratios for initial and final pass models. Points represent median values and vertical bands represent interquartile ranges (IQRs). Horizontal dashed lines indicate a perfect model fit.

We evaluated goodness‐of‐fit metrics for ARCHI models on the full NY and CA datasets to assess their relative ability to simulate the observed data (Figure 5). Modeled values for non‐imputed datapoints are not returned by the missForest algorithm, so it is not possible to evaluate corresponding goodness‐of‐fit metrics (i.e., simulated versus observed values). We compared goodness‐of‐fit metrics between the imputation algorithm's initial and final passes (Figure 2) using the Wilcoxon rank sum test evaluated at a 95% confidence level (R Core Team 2021).

Both OLS and ridge were able to fit regression models to targets from both datasets with median NSE values exceeding 0.80 and 0.90 for NY and CA datasets, respectively (Figure 5a and 5b). The MOVE.1 model performed somewhat comparably on the CA dataset but did not fit as well to targets from the NY dataset (median NSEs of 0.69 and 0.83 for initial and final pass models, respectively). Because the MOVE.1 model in its current implementation can only utilize one predictor, it is less flexible in its capacity to fit to complex time series than the multiple regression models (OLS and ridge). However, MOVE.1 was able to consistently preserve the variance of target records, as evidenced by variance ratios of unity. The OLS and ridge models indicated variance loss for both datasets, with reductions in target variance typically exceeding 5% and 10%, respectively (Figure 5c and 5d). The ridge model is specifically designed to reduce model variance to prevent overfitting, leading to more generalizable results in exchange for a slight increase in bias (Hastie 2020).

Generally, model fits improved from the initial to the final pass when models are re‐fit to the entire imputed dataset. Median NSEs were significantly greater for final compared to initial passes for all three considered regression models (Wilcoxon rank sum test *p*‐values <0.05; Figure 5a and 5b). Median variance ratios were also significantly greater for final compared to initial passes for OLS and ridge models (Wilcoxon rank sum test *p*‐values <0.05), but not MOVE.1 because they were unity in both cases (Figure 5c and 5d).

### Benchmarking

We used ARCHI to create 200 holdout datasets for each of the two input datasets by random removal of 5% of observed values (i.e., about 2300 and 3300 observations were removed per holdout dataset for NY and CA, respectively). Determination of the optimal proportion of holdout data is dataset‐specific, but we recommend evaluating repeated (*n* > 100) holdouts of small proportions (e.g., 5%) to provide a statistically robust subsample without perturbing the overall structure of the dataset. Here, we comparatively evaluated relative imputation performance by computing errors of withheld observations compared to imputed values across all four models (MOVE.1, OLS, ridge, missForest) for each holdout dataset. We compared resultant NRMSEs across all four groups using Dunn's all‐pairs rank comparison test with Benjamini‐Hochberg adjusted *p*‐values evaluated at a 95% confidence level (Pohlert 2021).

We further computed the coverage rates of bootstrapped 95% prediction intervals for all holdout datasets to evaluate the inferential capacity of ARCHI models. Ideal coverage rates for 95% prediction intervals are ≥0.95 and those falling below 0.90 indicate poor model inference (van Buuren 2018). The missForest algorithm computes dataset‐ and variable‐wide OOB errors but does not provide point‐wise error estimates to evaluate coverage rates. The missForest model produced OOB NRMSEs of 0.028 and 0.213 for NY and CA datasets, respectively, which were slightly higher than corresponding median holdout errors of 0.022 and 0.201 (Table 2).

**Table 2:** Model Results for 200 Random Holdout Datasets Evaluated Using 5% of the Original Observation Data.

Dataset	Model	Average Model Runtime, in Seconds	Median NRMSE (Interquartile Range)	Model NRMSE Comparison (Dunn Test)^1^	Average Model Runtime with Bootstrapping (*B* = 500), in Seconds	Median Coverage Rate for 95% Prediction Interval (Interquartile Range)
Long Island, NY	MOVE.1	3	0.033 (0.032–0.034)	MOVE.1 > OLS > **ridge, missForest**	127	0.91 (0.90–0.91)
	OLS	4	0.024 (0.023–0.026)		627	0.89 (0.88–0.89)
	ridge	75	**0.022 (0.021–0.023)**		2998	0.92 (0.92–0.93)
	missForest	192	**0.022 (0.021–0.023)**		NA	NA
Central Valley, CA	MOVE.1	90	0.231 (0.219–0.243)	MOVE.1 > missForest > OLS > **ridge**	748	0.88 (0.88–0.89)
	OLS	139	0.194 (0.185–0.209)		1397	0.92 (0.92–0.92)
	ridge	301	**0.181 (0.174–0.193)**		2128	0.93 (0.93–0.93)
	missForest	1134	0.201 (0.193–0.211)		NA	NA

Notes: Models with lowest NRMSEs for each dataset are shown in bold. NA indicates value not available. Runtimes are for a Dell Latitude laptop equipped with a 12th Gen Intel Core i9‐12950HX processor and 64 gigabytes of RAM.

1 How to read results for significant differences; “MOVE.1 > OLS > ridge, missForest” signifies NRMSE values for MOVE.1 models were significantly greater than those of OLS, which were significantly greater than those of ridge and missForest.

**Figure 6:**
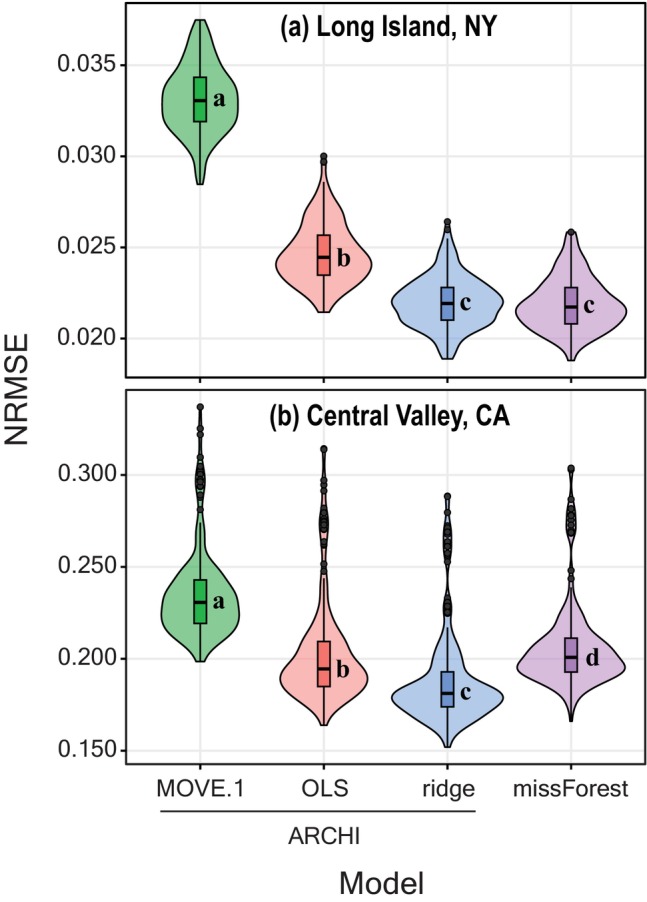
Combined violin‐boxplots showing model NRMSEs for 200 random holdout subsets for the (a) NY and (b) CA datasets, each evaluated using 5% of the original observation data (Levy 2024). Violin plots are shown with compact letter display, which indicates statistical difference between model error rates at a 95% confidence level for pairs containing different letter groups.

We found that runtimes for the three ARCHI models were faster than missForest by factors ranging from 2.6 to 64 (Table 2). Imputation runtimes for ARCHI generally scale with number of target sites, as evidenced by factor increases in runtimes ranging from 4.0 to 35 on the CA compared to NY datasets (1394 and 137 sites, respectively; Table 2). The NY dataset tended to have lower error rates than the CA dataset with median NRMSEs for all models ranging from 0.022 to 0.033 and 0.181 to 0.231, respectively (Table 2 and Figure 6). The CA records are more variable than the NY records (Table 1) due to intensive pumping during frequent droughts, which have caused substantial (tens of meters) declines in groundwater‐levels across the region (Levy et al. 2021). The lower accuracy of CA dataset imputations across all models could be due to the challenging structure of the dataset, which required a greater degree of extrapolation and extension beyond the range of the training data than the NY dataset (Table 1). Much of the extrapolation occurred near the end of the study period when groundwater levels reached historic lows (Figure 1).

The ridge model showed no significant difference or lower NRMSEs compared to those of missForest on respective NY and CA datasets. The OLS model had higher and lower NRMSEs compared to missForest on respective NY and CA datasets. The MOVE.1 model had higher NRMSEs than all other models on both datasets (Table 2 and Figure 6). Bootstrapping prediction intervals with B = 500 increased ARCHI model runtimes by factors ranging from 7 to 157. The median coverage rates for ARCHI prediction intervals were all <0.95, indicating truncation of prediction intervals from ideal ranges. The ridge model had the best coverage rates, with median values of 0.92 and 0.93 for NY and CA datasets, respectively. The OLS and MOVE.1 models had coverage rates <0.90 for NY and CA datasets, respectively, indicating poor inferential power (van Buuren 2018; Table 2).

Coverage rates below expected values are indicative of overly optimistic model precision. This likely occurs here due to effects of “data leakage” when a model is trained using a reference that contains information derived from the target (Kaufman et al. 2011). Data leakage can occur in ARCHI if initial models are overwritten during the final pass using references containing imputations derived from the target record. This can lead to artificial shrinkage of residuals and overoptimistic model fits with truncated prediction intervals. We tested this assumption by turning off the optional “final_pass” setting and found that median coverage rates for all models improved to 0.96 and 0.92–0.94 for NY and CA datasets, respectively (Table S1). However, turning off the final pass also significantly increased holdout NRMSEs for both datasets across all ARCHI models (Wilcoxon rank sum test *p*‐values <0.05; Table S1). Unlike overfit models, which are less generalizable to holdout data as fits improve (Figure 4b), final pass models show improved performance on both training and holdout data (Figure 5 and Table S1). The ridge model appears to be somewhat resistant to the effects of data leakage on model inference due to regularization.

## Model Applications and Limitations

The ARCHI package provides a generalizable workflow for processing and imputing large, hydrologic datasets where regional climatic drivers result in time‐series records exhibiting high degrees of multicollinearity. We found ARCHI's ridge regression setting had the lowest holdout NRSMEs and highest coverage rates on two contrasting groundwater‐level example datasets. The MOVE.1 setting preserved the variance of the target records but had the lowest imputation accuracies. The OLS setting provided a balance between imputation accuracy and variance loss, which was most pronounced for records imputed by ridge regression (Figures 5 and 6). The MOVE.1 and OLS settings may be better suited for applications where maintenance of target variance is of high priority, such as extreme value analysis.

Groundwater datasets imputed using ARCHI can be used in a variety of applications such as quantifying aquifer‐storage changes (Evans et al. 2020a), downscaling remote sensing data (Agarwal et al. 2023), and calibrating groundwater‐flow models (Faunt 2009). Although rigorous benchmarking of ARCHI imputation efficacy is restricted here to groundwater‐level time series, functions included in the package can be used broadly to format and visualize a variety of hydrologic datasets. For example, ARCHI provides functions to rapidly compile minimum, maximum, and user‐defined quantile of date‐indexed observation values on a variety of timescales, including user‐defined water years, which can be directly applied to peak‐over‐threshold and threshold‐level analyses often used to quantify hydrologic responses to flood and drought conditions (Brunner et al. 2021). Model analysis and validation tools provided by ARCHI can be applied to test the suitability of its approach for different types of hydrologic data. The open‐source and modular format of the ARCHI package is designed to accommodate additional regression models for specific applications. For example, future versions of ARCHI can include the MOVE.3 regression model (Hirsch 1982; Vogel and Stedinger 1985), which is more commonly utilized for imputing annual peak surface‐water flow records that exhibit minimal autocorrelation (Matalas and Jacobs 1964; Granato 2009; Siefken and McCarthy 2022).

The ARCHI approach inherently shares previously documented limitations of regression imputation, which can artificially strengthen correlations within a dataset (van Buuren 2018). Although, the intended application of ARCHI is on hydrologic records that already exhibit a high degree of cross‐correlation, idiosyncrasies in local records may be obscured by models trained on aggregate regional signals. Features driven by local dynamics may be better isolated by training models on a small number of reference sites (e.g., 5–10) or applying geospatial cutoff filters (Figure 3). Applying additional filters for reference selection may improve validation errors, in some cases, up to the point where they become too restrictive (e.g., Feng et al. 2014).

Fundamentally, the regression imputation method employed by ARCHI is limited by the availability of suitable references and will underperform if synchronous, network‐scale data gaps occur during a specific period. Such systemic data gaps may occur in hydrologic records due to widespread sensor failures during extreme events such as floods or droughts. Additional care should be taken if extending records in a large monitoring network to a historical period with comparatively fewer reference sites. Further, artifacts of poor data quality such as outliers or sensor drift may be propagated through datasets by ARCHI imputations and rigorous quality control of input data is of critical importance. Graphical tools included in the ARCHI package can help to identify specific gap‐pattern and data‐quality issues that may result in spurious imputations.

ARCHI may underestimate the width of prediction intervals in certain cases of data leakage, which may lead to overoptimistic model fits and poor inference. This effect can be curtailed by turning off the final pass setting and eliminated altogether by additionally setting the relax parameter to 0. In such cases, complete records are required to seed initial models and all targets are imputed only once. However, imputation accuracy may be reduced as a result (Table S1). The effects of the final pass setting on model inference can be tested by computing coverage rates for prediction intervals using the “hold_grid()” and “hold_eval()” functions as described above. The final pass setting can also diminish interpretability of reference selection as the initial most correlated references may be overwritten with those containing a large portion of imputed data derived from the target.

Although ARCHI provides prediction intervals to assess model uncertainty, it is a single imputation model and inherently limited if pooling is desired for statistical applications, as is standard practice for multiply imputed datasets (van Buuren 2018; Little and Rubin 2019). However, the popular multiple imputation programs MICE and Amelia are not overtly designed to process high‐dimensional datasets with strong multicollinearity. We attempted to apply these methods to our example datasets and found an arbitrary synthetic ridge prior had to be invoked for Amelia to allow inversion of multicollinear matrices. Additionally, the MICE algorithm becomes computationally inefficient for high‐dimensional datasets with hundreds of variables (van Buuren 2018) and was not able to impute the CA dataset in a reasonable timeframe (only 2% of the first model iteration was completed within 24 h using default settings). The ARCHI program implements automated variable selection and a sophisticated method of ridge regularization to handle high‐dimensional, multicollinear datasets in a computationally efficient manner. Future versions of ARCHI can be modified to incorporate multiple imputation by generating random initial estimates of NAs prior to regression during the initialization step of the “impute_grid()” algorithm.

## Summary and Conclusions

Here, we present a new R package, ARCHI, designed for flexible imputation of regionally correlated hydrologic records with benchmarked applications to large, groundwater datasets. We highlight ARCHI's computational efficiency, imputation accuracy, and model transparency in the context of other widely used multivariate imputation algorithms. We provide some basic guidelines for initial parametrization of ARCHI models, but suggest users leverage the included GUI for interactive exploration of different parameter combinations in real time. In addition to the primary application of missing‐data imputation, graphical tools included with the ARCHI package allow users to explore the correlation structure of regional hydrologic records. The open‐source and modular framework of the ARCHI package allows for maintenance of dependencies and contributions by a broad user base.

## Authors' Note

The authors do not have any conflicts of interest or financial disclosures to report.

## Supporting information


**Figure S1.** Histograms of groundwater‐level measurements by calendar month for example datasets.
**Figure S2.** Simulation of effects of data missingness proportions and patterns on model error for selected sites from example datasets.
**Figure S3.** Percentage of target sites imputed and mean fitted Nash‐Sutcliffe efficiency (NSE) as a function of model error threshold for example datasets.
**Table S1.** Comparison of final and initial pass models.

## Data Availability

The data that support the findings of this study are openly available in ScienceBase at https://doi.org/10.5066/P17NXGHV.
