# Supplementary material for: ARCHI: A New R Package for Automated Imputation of Regionally Correlated Hydrologic Records
Source: Ground Water. 2025 Feb 28;63(4):595–610. doi: 10.1111/gwat.13474 (PMC12272003; doi:10.1111/gwat.13474)
Supplement: Supplementary file 1 — Figure S1. Histograms of groundwater‐level measurements by calendar month for example datasets. Figure S2. Simulation of effects of data missingness proportions and patterns on model error for selected sites from example datasets. Figure S3. Percentage of target sites imputed and mean fitted Nash‐Sutcliffe efficiency (NSE) as a function of model error threshold for example datasets. Table S1. Comparison of final and initial pass models. [file GWAT-63-595-s001.docx]

Supporting Information for:

**ARCHI: A new R package for automated imputation of regionally correlated hydrologic data**

Levy, Z.F.^1^, Glas, R.L.^2^, Stagnitta, T.J.^2^, andTerry, N.^2^

^1^ U.S. Geological Survey, California Water Science Center, 6000 J Street, Placer Hall, Sacramento, California, 95819-6129, USA

^2^ U.S. Geological Survey, New York Water Science Center, 425 Jordan Road, Troy, NY, 12180, USA

**Contents of this file:**

Figures S1–S3

Table S1

**
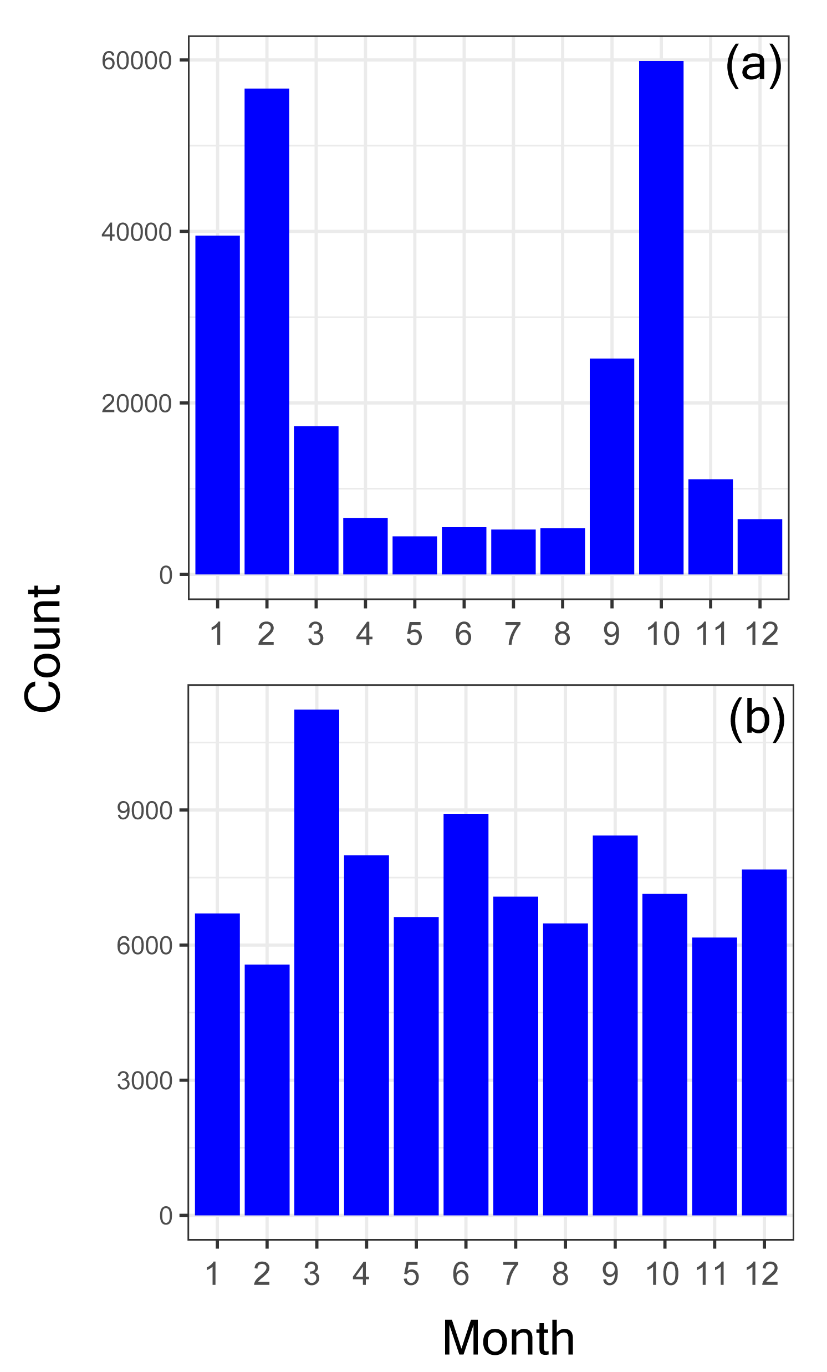
**

**Figure S1.** Histograms of groundwater-level measurements by calendar month in (a) CA and (b) NY datasets.

**
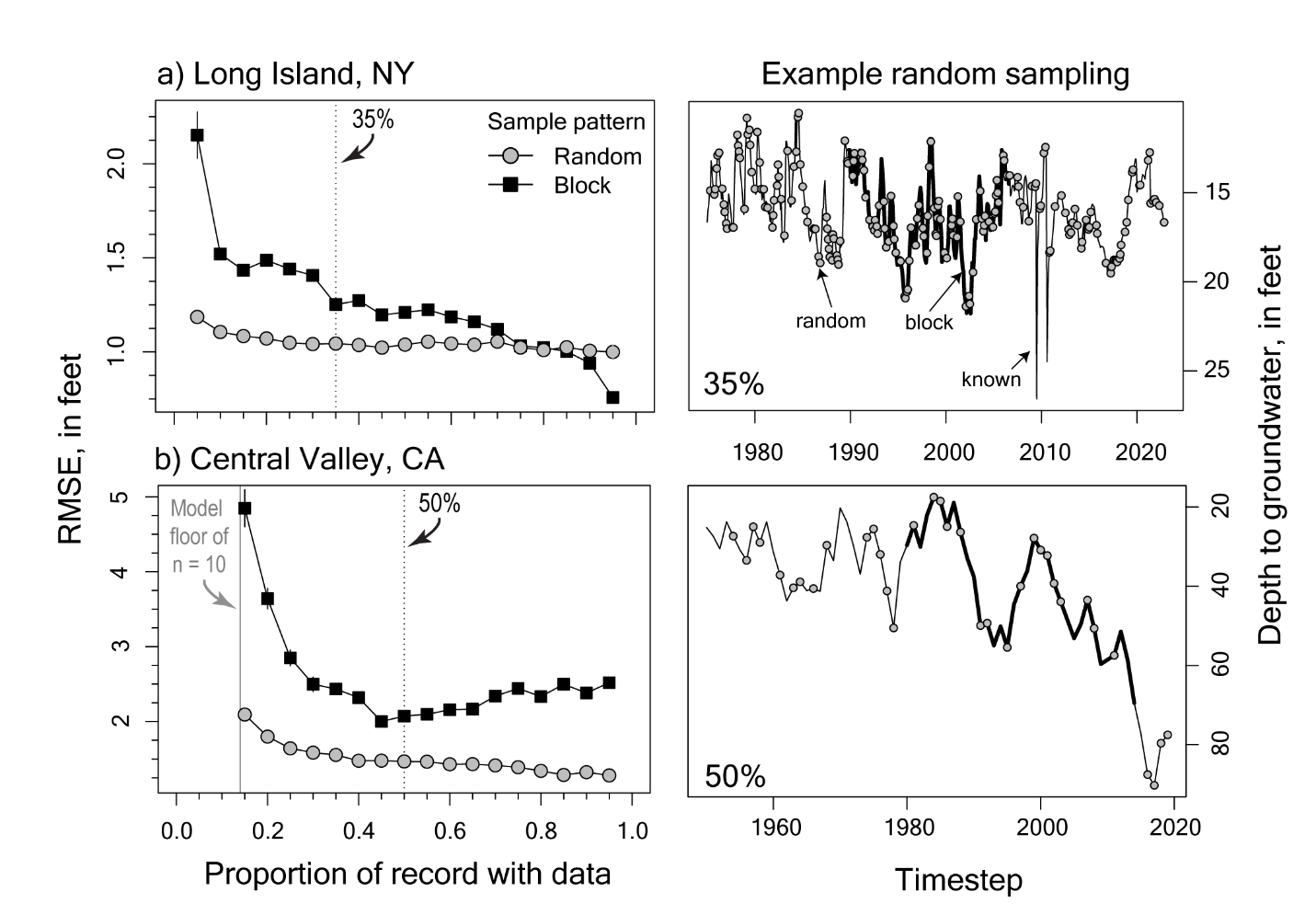
**

**Figure S2.** Simulation of effects of data missingness proportions and patterns on model error for example time series for (a) Long Island, NY and (b) Central Valley, CA datasets. An example record with near complete time series was selected for simulation from each dataset (site numbers of 404301073240904 and 365469N1196471W001 for NY and CA datasets, respectively, as detailed in Levy, 2024). Small gaps were imputed with the R “imputeTS” package using a Kalman filter with the na_kalman function (Moritz and Bartz-Beielstein, 2017) to create complete records. Data was sampled from the “known” records (thin continuous line in right panels) in random or continuous block patterns at different proportions. An example of one iteration of this random sampling at proportions of 0.35 and 0.50 are included in the right panels. This experiment was repeated for each site 200 times for each of the two sample patterns. Root mean square error (RMSE) was computed for each sampling by comparing imputed values to the non-sampled portions of the time series. Average RMSEs are shown for the different tested sampling proportions and patterns in the left-hand panels for the NY (top) and CA (bottom) test sites with standard error bars. In most cases standard error bars were smaller than the plotted point size. This may aid in choosing the `data_thresh` input to the `trim_grid()` function, approximated here as the elbows of respective error curves at 0.35 and 0.5 for NY and CA datasets, respectively.


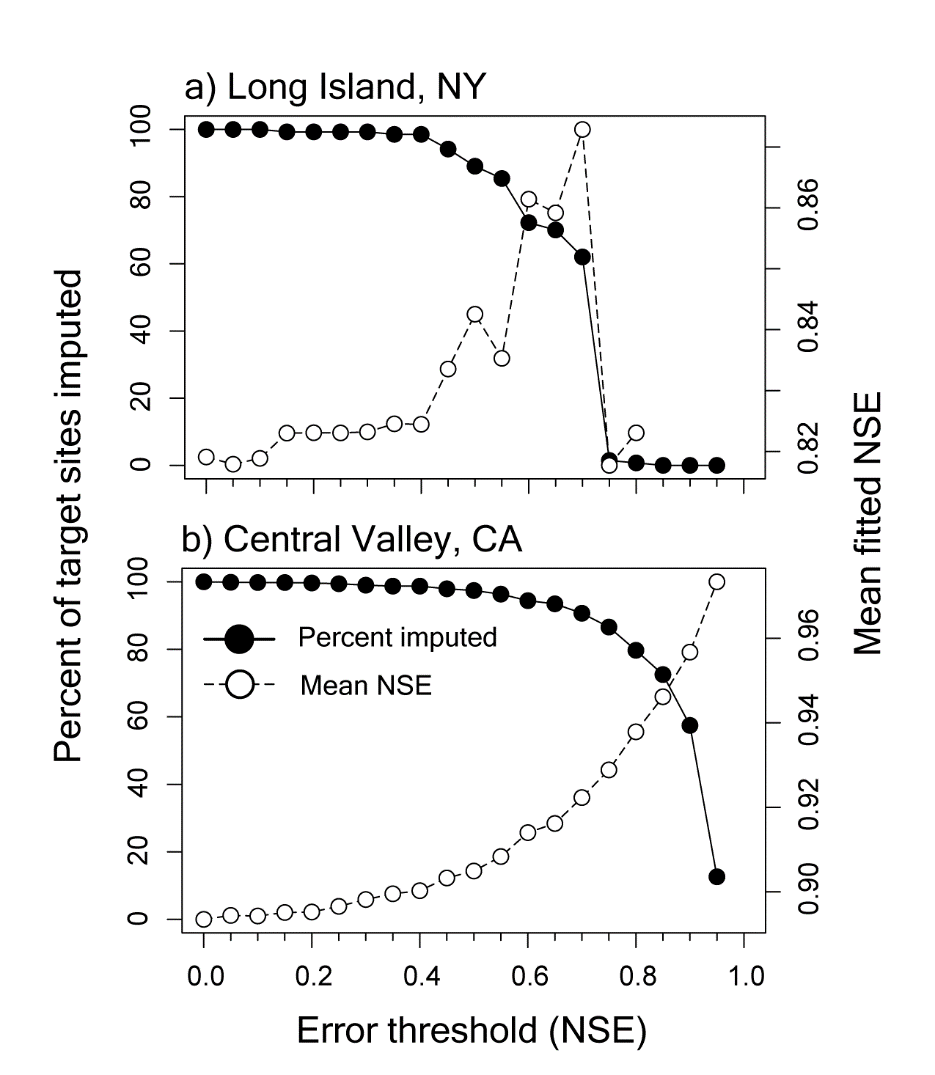


**Figure S3.** Percentage of target sites imputed and mean fitted Nash-Sutcliffe efficiency (NSE) as a function of model error threshold for (a) NY and (b) CA datasets. The ARCHI ridge model was used in all cases with n_refwl = “max” and p_per_n = 0.5.

**Table S1.** Comparison of final and initial pass models.

| **Dataset** | **Model** | **Median NRMSE for initial pass (intequartile range)** | **Median NRMSE for final pass (intequartile range)** | **Final pass NRMSE comparison, Wilcoxon rank-sum test result*** | **Median coverage rate for 95% prediction interval, initial pass (interquartile range)** | **Median coverage rate for 95% prediction interval, final pass (interquartile range)** |
| --- | --- | --- | --- | --- | --- | --- |
| Long Island, NY | MOVE.1 | 0.036 (0.034 – 0.037) | 0.033 (0.032 – 0.034) | Final_pass < Initial_pass | 0.96 (0.95 – 0.96) | 0.91 (0.90 – 0.91) |
|  | OLS | 0.027 (0.026 – 0.028) | 0.024 (0.023 – 0.026) | Final_pass < Initial_pass | 0.96 (0.96 – 0.97) | 0.89 (0.88 – 0.89) |
|  | ridge | 0.026 (0.026 – 0.027) | 0.022 (0.021 – 0.023) | Final_pass < Initial_pass | 0.96 (0.96 – 0.96) | 0.92 (0.92 – 0.93) |
| Central Valley, CA | MOVE.1 | 0.234 (0.224 – 0.246) | 0.231 (0.219 – 0.243) | Final_pass < Initial_pass | 0.92 (0.92 – 0.92) | 0.88 (0.88 – 0.89) |
|  | OLS | 0.201 (0.192 – 0.215) | 0.194 (0.185 – 0.209) | Final_pass < Initial_pass | 0.94 (0.94 – 0.95) | 0.92 (0.92 – 0.92) |
|  | ridge | 0.189 (0.181 – 0.199) | 0.181 (0.174 – 0.193) | Final_pass < Initial_pass | 0.94 (0.94 – 0.94) | 0.93 (0.93 – 0.93) |

*Note: How to read results for significant differences; “Final_pass < Initial_pass” signifies variance-normalized root mean square errors (NRMSEs) were lower for final pass compared to initial pass models.

**References**

Levy, Z.F., 2024, Example Groundwater-Level Datasets and Benchmarking Results for the Automated Regional Correlation Analysis for Hydrologic Record Imputation (ARCHI) Software Package. *U.S. Geological Survey Data Release,* <https://doi.org/10.5066/P17NXGHV>.

Moritz, S. and Bartz-Beielstein, T., 2017, imputeTS: Time Series Missing Value Imputation in R. R Journal: 9(1), <https://doi.org/10.32614/RJ-2017-009>.

**Disclaimer**

Any use of trade, firm, or product names is for descriptive purposes only and does not imply endorsement by the U.S. Government.
